# Effects of Acute Exercise Duration on the Inhibition Aspect of Executive Function in Late Middle-Aged Adults

**DOI:** 10.3389/fnagi.2019.00227

**Published:** 2019-09-03

**Authors:** Yu-Kai Chang, Feng-Tzu Chen, Garry Kuan, Gao-Xia Wei, Chien-Heng Chu, Jun Yan, Ai-Guo Chen, Tsung-Min Hung

**Affiliations:** ^1^Department of Physical Education, National Taiwan Normal University, Taipei, Taiwan; ^2^Institute for Research Excellence in Learning Science, National Taiwan Normal University, Taipei, Taiwan; ^3^Exercise and Sports Science, School of Health Sciences, Universiti Sains Malaysia, Kelantan, Malaysia; ^4^Key Laboratory of Behavioral Science, Institute of Psychology, Chinese Academy of Sciences, Beijing, China; ^5^College of Physical Education, Yangzhou University, Yangzhou, China

**Keywords:** acute exercise, dose-response relationship, executive control, exercise prescription, Stroop test

## Abstract

**Objective**: This study investigated whether acute exercise duration affects inhibition in late middle-aged adults.

**Methods**: Over four separate days, 40 late middle-aged adults completed, in a counterbalanced order, three exercise sessions consisting of single bouts of moderate-to-vigorous intensity cycling, with the main acute exercise durations being 10, 20, and 45 min, and a control session consisting of 30 min of reading. Their inhibition performance was then evaluated by administration of the Stroop test following each session.

**Results**: The participants had shorter mean response times for both the congruent and neutral conditions of the Stroop following the acute exercise lasting 20 min than they did after the control session. The acute exercise lasting 20 min also resulted in shorter response times for both conditions of the Stroop than the acute exercise lasting only 10 min. Meanwhile, the acute exercise lasting 45 min resulted in a shorter mean response time for the neutral Stroop condition than did the control session. Finally, the acute exercise lasting 20 min resulted in the shortest mean response time of all four sessions for the Stroop incongruent condition.

**Conclusion**: The above findings suggest that the moderate-to-vigorous intensity acute exercise lasting 20 min facilitated multiple cognitive function domains in general, whereas the exercise sessions of shorter and longer duration had negligible effects on executive function in the late middle-aged adults. These results highlight the need to consider the duration of any moderate-to-vigorous intensity exercise when developing acute exercise programs to facilitate executive function in aged populations.

## Introduction

Individuals in the later stages of life frequently experience deterioration in numerous cognitive function domains, including processing speed, working memory, and long-term memory (Park and Reuter-Lorenz, 2009), with executive function having been emphasized by researchers as a type of high-order cognition (or meta-cognition) that is particularly affected in old age (Adólfsdóttir et al., 2017; Fjell et al., 2017). As individuals age beyond a certain point, they experience significant gray matter reductions in the prefrontal cortex, as well as age-related deficits in the functional hyperactivation aspect of executive function, suggesting that the reductions in executive function and brain volume are reciprocal and associated with aging (Di et al., 2014). Notably, executive function and its associated brain structures can be altered by various factors including exercise, which can also affect the extent to which the function and structures adapt to the effects of aging (Prado Lima et al., 2018; Chen et al., 2019).

Acute exercise, defined as a single bout of exercise, has been shown to have a positive relationship with executive function (Audiffren and André, 2019; Etnier and Chang, 2019). A meta-analysis conducted by Chang et al. (2012) revealed that acute exercise facilitates executive function, regardless of whether the said function is assessed immediately after the termination of the exercise or after a delay following the termination of the exercise. The benefits of acute exercise on cognitive function are disproportional (Hillman et al., 2014; Li et al., 2014). Studies employing the Stroop test have shown that acute exercise can lead to positive impacts on the performance of both the incongruent and congruent conditions, as well as the neutral condition, of the test (Chang et al., 2015a; Chang Y. K. et al., 2017; Chu et al., 2017). Given that the Stroop test simultaneously measures inhibition, one of three primary subcomponents of executive function, through its incongruent condition and related basic information processing through its other two conditions (Miyake et al., 2000) these findings suggest that acute exercise facilitates multiple cognitive functions in general, including the inhibition aspect of executive function.

Several factors should be considered in order to maximize the effects of acute exercise on executive function. For example, it has been proposed that exercise intensity can modulate the relationship between acute exercise and executive function. Wang et al. (2016) reported that although the inhibitory control performance of study participants was improved following acute aerobic exercise, there was an inverted-U trend in the relationship between exercise intensity and inhibition, with moderate intensity exercise sessions resulting in larger beneficial effects than light intensity and control sessions. Similar inverted-U trends were also observed with different modes of exercise. Chang and colleagues indicated that acute resistance exercise at moderate intensity [70% of a 10-repetition maximum (RM)] resulted in better executive control performances in terms of both inhibition (Chang and Etnier, 2009) and planning (Chang et al., 2011) than light (40% of 10-RM) and vigorous (100% of 10-RM) sessions of acute resistance exercise. These findings suggest that moderate intensity exercise may be preferable when designing acute exercise programs to ameliorate executive function.

The relationship between executive function and exercise duration, another major component in the design of acute exercise programs, has been less frequently examined. In fact, to the best of our knowledge, only two studies have investigated the relationship. To identify the effects of different exercise durations, Chang et al. (2015b) had college-aged individuals participate in sessions with four different durations of moderate-to-vigorous intensity exercise (i.e., 0 min, 10 min, 20 min, and 45 min) and then complete the Stroop test. An inverted-U trend was demonstrated, with the participants who participated in the 20-min session showing the best cognitive performances in the Stroop congruent and incongruent conditions. Interestingly, however, the effects of exercise duration on executive function may be age-dependent. Utilizing a similar exercise protocol, a follow-up study showed both linear and cubic trends in the relationship between exercise duration and the task switching aspect of executive function in late middle-aged older adults, suggesting that while 20 min of acute moderate-to-vigorous intensity exercise is an effective duration, the longer duration does not necessarily harm executive function in late middle-aged adults (Chen et al., 2018). However, whether these findings extend to the inhibition aspect of executive function in late middle-aged adults remains unknown.

The purpose of the present study was to address this knowledge gap by determining the relationship between exercise duration and the inhibition aspect of executive function in late middle-aged adults. Specifically, the acute effects of three durations of moderate-to-vigorous intensity exercise (i.e., 10 min, 20 min, and 45 min) on inhibition as assessed by the Stroop test in late middle-aged adults were compared. It was hypothesized that the 20-min duration would show the greatest benefits in terms of inhibitory performances in this specific population.

## Materials and Methods

### Participants

Forty late middle-aged adults (24 females; *M*_age_ = 57.58 ± 4.90 years) from the cities of Taoyuan and Taipei in Taiwan were recruited. This sample size met the minimum requirement for identifying the acute exercise effects on executive function G-Power alpha = 0.05, power = 0.8, and effect size as partial *η*^2^ = 0.16 (Chang et al., 2015b). All of the enrolled participants met the following inclusion criteria: (1) age of 55–65 years old; (2) Mini-Mental State Examination (MMSE) score greater than 26; (3) Right hand dominant; (4) Normal vision and no color blindness; (5) No medical conditions involving neurological or psychiatric disorders; and (6) Low-risk status, indicating freedom from any conditions making participation in acute exercise inappropriate, according to the Physical Activity Readiness Questionnaire (PAR-Q; Liou et al., 2008). All participants were informed of the potential risks and discomforts associated with participation in the study. In order to measure working memory as a potential covariant, the Digit Span test of the Wechsler Adult Intelligence Scale—Third Edition (Wechsler, 1997) was administered. All of the participants completed a written informed consent form, and the study was approved of by the Institutional Review Board of Fu-Jen Catholic University. The recruitment protocol replicated that of a previous study (Chang et al., 2015a) and followed the guidelines of the American College of Sports Medicine (American College of Sports Medicine, 2013). The participants’ demographic characteristics are presented in Table 1.

**Table 1:** Participant characteristics (M ± SD).

Variable	Male	Female	Total
Sample size	16 (40%)	24 (60%)	40 (100%)
Age (years)	58.56 ± 5.28	56.92 ± 4.62	57.58 ± 4.90
Height (cm)	171.81 ± 3.97	156.90 ± 4.62	162.86 ± 8.54
Weight (kg)	75.50 ± 9.18	56.58 ± 8.53	64.15 ± 12.78
BMI (kg.m^−2^)	25.46 ± 2.43	22.95 ± 3.42	23.95 ± 3.27
Education (years)	15.47 ± 1.92	13.25 ± 2.33	14.10 ± 2.41
MMSE	28.31 ± 1.78	19.87 ± 4.61	20.43 ± 4.29
Digit span-forward	15.25 ± 1.00	14.42 ± 2.39	14.75 ± 1.98
Digit span-backward	6.00 ± 2.97	5.46 ± 2.87	5.68 ± 2.89
Resting HR	68.56 ± 7.38	72.50 ± 9.46	70.84 ± 8.76
VO_2peak_ (mL.kg^−1^.min^−1^)	35.53 ± 11.42	38.15 ± 8.85	37.10 ± 9.88

*Notes: M, mean; SD, standard deviation; BMI, body mass index*.

### The Stroop Test

Two types of cognitive function tests, the Stroop test and the task-switching test, were administered. The present study focused on the Stroop test and specific details of task-switching, as another aspect of executive function, presented in previous studies (Chang Y. K. et al., 2017; Song et al., 2017; Chen et al., 2018).

The Stroop test (Stroop, 1935), a widely used neuropsychological assessment of the inhibition aspect of executive function, has previously been employed in various studies of acute exercise (Vasques et al., 2011; Hwang et al., 2016; Chang E. C. H. et al., 2017; Chu et al., 2017; Lowe et al., 2017). In the computer version of the test, a total of four blocks with 60 trials in each block are presented. During each trial, one of three single-word stimuli (i.e., “blue,” “red,” or “green”) in Chinese is presented in the center of a computer screen on a white background (Neuroscan Stim Software), the distance of which was 70 cm away from the participants in the current study. Each of the aforementioned four blocks contains trials presenting the three types of Stroop conditions, namely, the congruent, neutral, and incongruent conditions. In a congruent condition trial, the word stimulus is displayed in the color matching the meaning of the word (e.g., the word “blue” is presented in blue color). In a neutral condition trial, the word stimulus is presented in a color that does not match it or the other two word stimuli (i.e., in black). In an incongruent condition trial, the word stimulus is displayed in a color matching the meaning of one of the other two word stimuli (i.e., the word “blue” is displayed in either red or green color). The rates at which the word stimuli and color match were designed and organized to minimize potential confounds (e.g., the number of stimuli, word facilitation, etc.). Those taking the test are instructed to perform the test as quickly and accurately as possible. The total testing duration is roughly 20 min, with 2-min rest intervals between the four blocks. Both the response time and response accuracy for accurate trials were elicited as the primary indices for further analysis in this study.

### Cardiorespiratory Fitness Assessment

The YMCA cycle ergometry protocol (Golding, 1989), a submaximal cardiovascular test, was individually administered to directly determine the VO_2peak_ for each participant. The protocol was designed for adults in the Class A risk stratification (Fletcher et al., 2001), and by completing three consecutive 3-min cycling stages, each participant’s fitness level was calculated. The initial cycling stage, which requires pedaling at a speed of 150 kpm·min^−1^ or 0.5 kg, was set up to maintain the workload continuously for 3 min, and the experimenters recorded each participant’s heart rate during the final 15–30 s of the 2nd and 3rd min. These two HRs for the first stage were then utilized to determine the workload in the second and third stages. For example, participants with a HR of less than 80 bpm in the first stage were then given a workload of 125W (750 kpm/min) for the second stage and 150W (900 kpm/min) for the third stage. The experimenters then analyzed the performances once the participants had reached the target HR for each consecutive stage, and the submaximal cardiovascular index, VO_2peak_, of each participant was calculated by adding the reference of individual body weight and age-predicted heart rate [206 − (0.67 × age); Gellish et al., 2007].

### Exercise Intensity Manipulation Check

#### Heart Rate

Each participant was asked to wear a short-range radio telemetry device (Sport Tester PE 3000, Polar Electro Oy, Kempele, Finland) to monitor his or her heart rate throughout the treatment sessions. Two heart rate variables were elicited: HR_rest_, the heart rate at the baseline of each session, and Hd_during_, the averaged heart rate recorded at the 2-min intervals during each session.

#### Rating of Perceived Exertion (RPE)

The rating of perceived exertion (RPE) scale was employed to assess each participant’s perceptions of his or her effort during the exercise sessions. The RPE scale was designed by Borg (1982) and has a range of a 6–20. Scores of 7–11 are regarded as indicating very, very light to fairly light effort; scores of 13–14 are regarded as indicating somewhat intense effort; scores of 15–19 are regarded as indicating intense to very, very intense effort; and a score of 20 is regarded as indicating exhaustion and maximal exertion. The experimenters recorded each participant’s score for the scale at the 2-min intervals of each session and then averaged the data in order to determine the RPE_during_.

### Procedure

A within-subjects repeated measure design was employed, in which all the participants visited the laboratory on four separate days in order to complete three exercise sessions (i.e., with the sessions lasting 20, 30, or 55 min) and a control session consisting of 30 min of reading. The participants were instructed to avoid caffeine 12 h prior to their visits to the laboratory (Cheng et al., 2016). In the exercise sessions, each participant was instructed to perform a single bout of cycling exercise at the initial cycling speed of 70 rpm, and then the resistance was gradually increased until 60%–70% of his or her heart rate reserve (HRR) was achieved. Each exercise session was designed following the recommendations of the ACSM, and thus consisted of three phases: a warm-up phase, main exercise phase, and cool-down phase (American College of Sports Medicine, 2013). Given 5 min each of warm up and cool down, then, the main exercise durations for the three exercise sessions were 10, 20, and 45 min. The control session required the participants to simply sit quietly while reading sports or exercise magazines for 30 min. For each session, each participant visited the laboratory at approximately the same time of day, with at least 3 days between each session. The participants were asked not to engage in any form of workout and/or exercise on the day of each session.

On the first visit of each participant, an informed consent form was completed by the participant, after which he or she completed a health history and demographics questionnaire and the PAR-Q. Each eligible participant then completed the Digit Span test. On the same day, the participant was randomly assigned into 1 of the 24 possible orders designated by a counterbalanced measures design to undergo the first treatment. At the beginning of each treatment, the given participant was asked to sit quietly in a dimly lit room for roughly 5–10 min for a test of his or her HR_rest_. Afterward, the participant was instructed to practice the Stroop test until 85% accuracy had been achieved for each of the blocks. One of four possible sessions was then begun. After the treatment was completed, the official Stroop test was administered to the participant. The process was similar for each of the other three sessions except for the treatment applied. Submaximal cardiovascular tests were also administered following the official Stroop test during the control session visit.

### Statistical Analysis

Two sets of analyses were conducted. A one-way repeated-measure analysis of variance (ANOVA) was employed to analyze the heart rate data for each of the treatment sessions (i.e., the control session and the 10-min, 20-min, and 45-min exercise sessions) in order to ensure the effectiveness of the exercise intensity manipulation. A two-way ANOVA with a 4 (treatment session: control session and 10-min, 20-min, and 45-min exercise sessions) × 3 (Stroop test condition: congruent, neutral, and incongruent) design was employed to analyze the response time and accuracy data, respectively. In the process of analyzing the data, Greenhouse-Geisser corrections were applied, and paired *t*-tests were conducted using Bonferroni adjustments for multiple comparisons. The effect sizes, using partial eta-squared, were reported for the significant main and interaction effects. An alpha of 0.05 was used for all statistical data.

## Results

### Exercise Manipulation Check

The heart rate and RPE data are summarized in Table 2. The one-way repeated ANOVA revealed that the three exercise sessions resulted in higher mean HR_during_ values than the control session, [*F*_(3,154)_ = 303.20, *p* < 0.001], with no significant difference observed among the three exercise sessions. With regard to RPE_during_, no significant differences were observed among the three exercise sessions, and the average scores ranged from 12 to 15.

**Table 2:** Descriptive data of exercise manipulation check (M ± SD).

Variable	Treatment session			
	Control	10 min	20 min	45 min
HR_average_	72.76 ± 8.13	126.36 ± 10.74	128.58 ± 10.43	128.01 ± 9.55
(beats^.^min^−1^)				
RPE_average_	N/A	13.06 ± 2.09	13.64 ± 1.86	13.64 ± 1.77

*Notes: M, mean; SD, standard deviation; HR_average_, heart rate assessed during each treatment session; RPE_average_, rating of perceived exertion assessed during each treatment session*.

### Stroop Test

Descriptive data for response time and accuracy are presented in Table 3.

**Table 3:** Response time and accuracy rate for stroop test following the exercise sessions (M ± SD).

Variable	Treatment session			
	Control	10 min	20 min	45 min
**Response time (ms)**				
Congruent	599.16 ± 57.56	589.24 ± 56.14	576.84 ± 53.36	589.30 ± 51.63
Neutral	612.04 ± 59.96	595.41 ± 56.45	576.12 ± 48.27	594.14 ± 54.50
Incongruent	659.88 ± 75.12	649.26 ± 70.64	629.28 ± 64.20	651.06 ± 66.70
**Accuracy rate (%)**				
Congruent	96.00 ± 6.88	96.39 ± 6.49	96.20 ± 6.72	96.63 ± 5.66
Neutral	96.03 ± 4.80	96.52 ± 4.58	96.61 ± 4.66	96.95 ± 3.64
Incongruent	93.10 ± 7.70	94.05 ± 7.37	94.29 ± 8.63	94.71 ± 6.53

*Notes: M, mean; SD, standard deviation*.

#### Response Time

A two-way repeated ANOVA revealed that there was a significant main effect for treatment session (*F*_(3,117)_ = 7.84, *p* < 0.001, partial *η*^2^ = 0.17), Stroop test condition (*F*_(2,78)_ = 161.98, *p* < 0.001, partial *η*^2^ = 0.81), and the interaction between treatment session and Stroop test condition (*F*_(6,234)_ = 2.57, *p* = 0.04, partial *η*^2^ = 0.06) on Stroop test response time.

**Figure 1:**
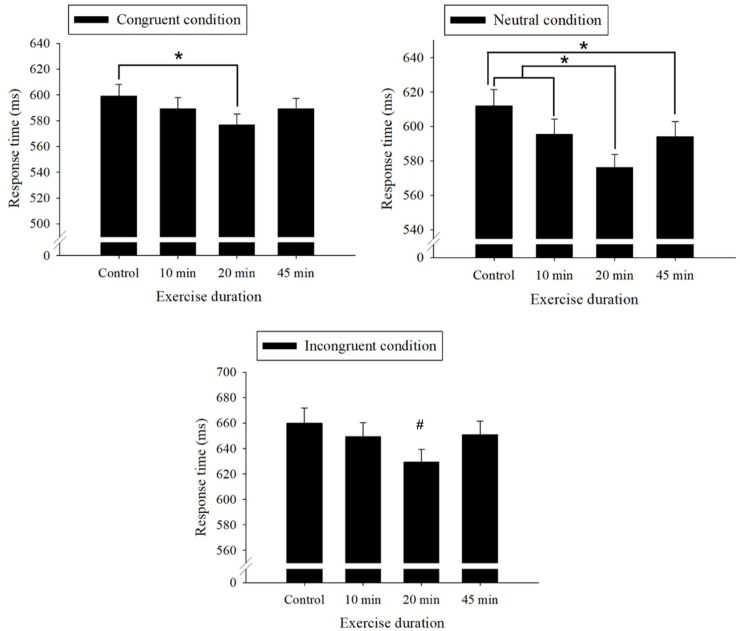
Stroop test performances (mean and standard error) stratified by treatment sessions. *Indicates a significant difference between the treatment sessions, *p* < 0.05. ^#^Indicates a significant difference between the 20-min treatment and the Control, 10-min and 45-min treatments.

The *post hoc* analysis that decomposed the interaction between treatment session and Stroop test condition revealed that the 20-min exercise treatment resulted in a shorter mean response time than the control treatment (*p* < 0.001) in the congruent condition. The 20-min treatment also resulted in a shorter response time than both the control session (*p* < 0.001) and the 10-min treatment (*p* = 0.03) for the neutral condition, while the 45-min treatment resulted in a shorter response time than the control session (*p* = 0.024) for the neutral condition. Last, the 20-min treatment resulted in a significantly shorter response time than the control session (*p* < 0.001), 10-min treatment (*p* = 0.05), and 45-min treatment (*p* = 0.02) in the incongruent condition (Figure 1).

#### Accuracy

A two-way repeated ANOVA revealed a main effect for task condition (*F*_(2,78)_ = 6.58, *p* = 0.01, partial *η*^2^ = 0.14), but no main effect for treatment session (*F*_(3,117)_ = 1.10, *p* = 0.35) or the interaction between treatment session and test condition (*F*_(6,234)_ = 0.64, *p* = 0.70). The pairwise comparisons for task condition revealed that accuracy rates for the congruent and neutral conditions were better than that for the incongruent condition (*p* < 0.001).

## Discussion

The present study was among the first to examine the effects of acute exercise on inhibition among late middle-aged adults by employing multiple exercise durations. The primary results revealed that the acute exercise with a duration of 20 min resulted in a shorter mean response time compared to the control session in the congruent condition of the Stroop test. Additionally, the 20-min and 45-min exercise durations also resulted in shorter response times than the control session for the neutral condition, with the 20-min duration also resulting in a shorter response time than the 10-min duration for the neutral condition. Last, the 20-min exercise duration resulted in a significantly shorter response time than the other three sessions for the incongruent condition. These improvements associated with acute exercise were accompanied by no significant change in accuracy, regardless of the Stroop test condition in question.

Moderate-to-vigorous intensity exercise with a main phase lasting 20 min is one of the frequently used exercise designs in studies of acute exercise and cognitive function (Ludyga et al., 2016). In accordance with our hypothesis, this duration of exercise elicited the optimal performance in all of the conditions of the Stroop test, suggesting that exercise generates a general improvement by improving both basic information processing (i.e., of the type used in the congruent and neutral conditions) and inhibition (i.e., which is used in the incongruent condition). With the longer response times and decreased accuracy rates for the incongruent condition compared to the congruent and neutral conditions, demonstrating the typical Stroop effect (MacLeod, 1991; Milham et al., 2002) as well as no difference in accuracy for the exercise sessions vs. the control session, these results suggest that our manipulation of the Stroop test was appropriate and that the improvements brought by the exercise consisted of faster responses while not resulting from a speed–accuracy trade-off. These results indicating improvement in two cognitive function domains, in general, are consistent with those of previous studies finding that exercise enhanced performance for both the congruent and incongruent conditions of the Stroop test, regardless of the individual’s fitness level, in older adults (Chu et al., 2015), as well as similar findings of general improvement in studies of exercise employing the Stroop test in young adults (Chang et al., 2015b; Chang Y. K. et al., 2017; Chu et al., 2017). The improved inhibition afforded by exercise is important because inhibition has been demonstrated to be impaired in aging populations (Hsieh and Lin, 2017). Taken together, the results of the present study replicate those of previous studies finding that 20 min of moderate intensity exercise facilitate multiple cognitive function domains including inhibition.

Although the better performance after the 20-min exercise session than the 10-min exercise session supported our hypothesis, the fact that the 10-min session failed to affect cognitive function was unexpected and inconsistent with previous studies finding that low-intensity exercise (30% of peak oxygen consumption) for 10 min improved Stroop test performance (Byun et al., 2014) and hippocampal memory function (Suwabe et al., 2018) in healthy young adults. While the difference may have been related to the intensity (e.g., moderate vs. light), tasks (e.g., memory), or population (e.g., younger adult) that these earlier studies considered, our results were nonetheless inconsistent with theirs. On the other hand, a meta-analysis by Chang et al. (2012) indicated that exercise sessions of less than 10 min in length could even result in negative impacts on cognitive performance. In addition, Chang et al. (2015b) also observed that exercise of less than 20 min in duration has limited effects on performance of both the congruent and incongruent conditions of the Stroop test in younger adults (Chang et al., 2015b) and task-switching in late middle-aged adults (Chen et al., 2018), when utilizing a dose-response relationship between exercise duration and executive function. Given those results and those of the present study, we suggest that exercise with moderate intensity for 10 min has negligible effects on inhibition in late middle-aged populations.

Meanwhile, the findings for the 45-min exercise session in this study showed an interesting pattern of better performance compared to the control session for the neutral condition and no difference for the incongruent condition, suggesting that the type of cognitive function in questions modulates the relationship between the exercise duration and cognitive performance. Specifically, the long exercise duration presented somewhat positive effects on basic information processing, whereas it had no apparent effect on the inhibition aspect of executive function. Given that only a few studies have examined longer exercise durations, however, a precise interpretation is not possible. That said, using a dose-response design to examine the relationship between exercise intensity and cognitive function (Chang and Etnier, 2009) found a specific improvement by showing a linear trend for a relatively simple task and an inverted-U trend for a task requiring a greater cognitive load, suggesting that longer exercise durations with a large exercise load facilitate basic information processing but not executive function. Additionally, Coles and Tomporowski (2008) reported that 40 min of submaximal aerobic exercise at 60% maximal capacity did not influence switching performance as compared to a control condition, suggesting that longer bouts of acute exercise have no effects on executive function. It should be noted that such findings of no additional benefits to executive function resulting from longer exercise durations are also consistent with the results of previous studies by Chang et al. (2015b) and Chen et al. (2018). Therefore, our findings suggest that exercise sessions of longer duration have specific influences on cognitive function, with no influence on the inhibition aspect of executive function in late middle-aged adults.

Physiological changes resulting from acute exercise have been proposed as an underlying mechanism of the benefits of such exercise to cognitive function (McMorris and Hale, 2012). Studies have observed an inverted-U trend in the relationship between exercise intensity and cognitive function when duration is controlled, with exercise for 20 min at moderate intensity resulting in better performance compared to exercise at light or high intensity (Chang et al., 2015b; Chen et al., 2018). Indeed, executive control is susceptible to exercise-induced arousal states (Byun et al., 2014), and these findings suggest that exercise induces optimal physiological changes to facilitate cognitive function. Additionally, a classic study using event-related potentials observed that acute exercise at moderate intensity for 20 min elicited brain activation by increasing P3 amplitudes and shortening P3 latencies during the performance of an executive control task (Hillman et al., 2003). Similar neuronal activation results associated with acute exercise have been replicated by numerous later studies (Hillman et al., 2012; Drollette et al., 2014; Chang et al., 2015a; Chang Y. K. et al., 2017). These changes in P3 indicate that acute exercise increases the allocation of attentional resources and the efficiency of information processing in executive control for a given task (Polich, 2012). It should be noted, however, that the mechanisms underlying these physiological and neuronal activation changes from shorter and longer durations of exercise remain unclear because no study to date has applied biological and/or neuroelectrical approaches to examine the relationship between exercise duration and executive function. Future studies of this sort are thus encouraged to further investigate and elucidate the effects of exercise with shorter and longer durations on executive function in late middle-aged adults.

Several limitations of the current investigation should be acknowledged. First, although the present study employed three exercise durations, in terms of intensity, only moderate intensity exercise was investigated. It is possible, however, that the optimal duration is intensity-dependent, and indeed, in order to determine the optimal effects of exercise on cognitive function, different intensities, durations, and modalities of exercise should simultaneously be considered (Chang et al., 2012). Additionally, the present investigation may have been restricted by only measuring the inhibition aspect of executive control. Etnier and Chang (2009) proposed that the impacts of exercise are dependent on different executive function domains and even the tasks that measure specific executive function domains. The generalization of the present study’s results should, therefore, be interpreted with caution. Last, any moderators that may affect the effects of exercise on cognition should be considered (Kato et al., 2018; Miller and Taylor-Piliae, 2018). While acute exercise improves cognitive function, the improvement is larger for individuals with higher fitness compared to those with low fitness (Chu et al., 2015) or for those with low-income status than those with high-income status (Tine, 2014), suggesting that the relationship could be modulated by personal and environment characteristics. Future studies are suggested to evaluate these possibilities.

## Conclusion

The importance of establishing a link between acute exercise and executive function in late middle-aged adults has already been recognized, but the efficacy of different exercise durations requires continued research. The present study provides empirical evidence that moderate-to-vigorous intensity exercise for 20 min, but not for shorter durations, is optimal for both basic information processing and executive function, while a longer duration of exercise may have positive effects on basic information processing but no beneficial effects on executive function. These findings provide the foundation for designing acute exercise programs with various exercise durations that could potentially facilitate different types of cognitive function in late middle-aged adults.

## Data Availability

The datasets generated for this study are available on request to the corresponding author.

## Ethics Statement

All of the participants completed a written informed consent form, and the study was approved of by the Institutional Review Board of Fu-Jen Catholic University.

## Author Contributions

A-GC, GK, and G-XW: formal analysis. JY, C-HC, T-MH, and Y-KC: data curation. T-MH and Y-KC: supervision. All authors: conceptualization, methodology, writing—original draft preparation and writing—review and editing.

## Conflict of Interest Statement

The authors declare that the research was conducted in the absence of any commercial or financial relationships that could be construed as a potential conflict of interest.
